# Caries prevalence and impact on oral health-related quality of life in children with sickle cell disease: cross-sectional study

**DOI:** 10.1186/s12903-015-0052-4

**Published:** 2015-06-18

**Authors:** Maria Luiza Matta Felisberto Fernandes, Ichiro Kawachi, Patrícia Corrêa-Faria, Marcos Pascoal Pattusi, Saul Martins Paiva, Isabela Almeida Pordeus

**Affiliations:** Department of Pediatric Dentistry and Orthodontics, Federal University of Minas Gerais, Av. Antônio Carlos, 6627, Faculdade de Odontologia, UFMG – Campus Universitário, 31270-901 Belo Horizonte, MG Brazil; Harvard School of Public Health, Department of Social and Behavioral Sciences, Boston, MA USA; Public Health Graduate Program, Vale do Rio dos Sinos University, São Leopoldo, RS Brazil

**Keywords:** Quality of life, Sickle cell disease, Dental caries, Socioeconomic factors

## Abstract

**Background:**

Children with sickle cell disease (SCD) may present oral conditions that can compromise children's health even more. However, there is still no consensus on the association between SCD and dental caries. The aim of this study was to assess caries prevalence in children with sickle cell disease (SCD), and the association of dental caries with socioeconomic factors, disease severity, and oral-health related to quality of life (OHRQoL).

**Methods:**

The sample was comprised of 106 children with SCD aged 8 to 14 years who were attending the Center for Hematology (Hemominas) in Belo Horizonte, Brazil. They were matched to 385 healthy peers. Data collection included interviews with guardians concerning SCD characteristics, and previous social and oral examinations to determine the caries prevalence. Caries prevalence as measured through the Decayed, Missing and Filled (dmft and DMFT) indices. OHRQoL was evaluated through the Brazilian versions of the Child Perceptions Questionnaires (CPQ_8–10_, and CPQ_11–14_ short-form version). Statistical analyses were performed using the chi-square test or Fisher`s exact test and the Mann Whitney test, as well as linear regression.

**Results:**

The DMFT index was 1.3 (SD: 2.1) in younger children with SCD and 1.5 (SD: 1.9) in SCD teens. Younger children with SCD had lower caries experience compared to healthy peers (*p* = .03). The experience of dental caries among teens with SCD was similar to healthy peers (*p* > 0.05). In addition, we did not see a significant difference on the mean overall scores of CPQ_8–10_ between SCD younger children and controls. There was no statistically significant difference in the mean overall scores of teens CPQ11-14 between SCD and the control group. Socioeconomic variables were not associated with dental caries in the participants with SCD. However, SCD severity was associated with higher DMFT indexes (*p* < 0.05).

**Conclusions:**

Younger children with SCD had a low experience of dental caries. The dental caries experience in teenagers with SCD was similar to their healthy peers. OHRQoL was similar between SCD participants and controls.

## Background

Sickle cell disease (SCD) is one of the most widespread genetic disorders worldwide, and the genotype (HbSS) is the most common and severe form of the disease [1]. Global estimates suggest that more than 312,000 infants are born with HbSS each year [2]. The United States and Europe together represent 2 % of annual SCD births worldwide [3]. The vast majority of sickle cell births occur in the developing world, with an estimated 230,000 annual HbSS births in sub-Saharan Africa [3]. Each year, 3,500 children in Brazil are born with SCD. Within Brazil, the state of Minas Gerais ranks third in prevalence of the disease [4].

The pathological effects of sickle cell disease also occur in dental tissues and the oral cavity. SCD has been linked to delayed tooth erosion, hypoplasia and hypomineralization, hypercementosis, pulp stones, and asymptomatic pulp necrosis due to thrombosis in the blood vessels [5]. There is no consensus regarding the caries experience in SCD patients compared to healthy controls. According to a study carried out with American teenagers, there was no difference in the caries experience among patients with SCD and their controls [6]. However, others studies related differences among individuals with SCD and without this disease [7, 8]. The greater occurrence of dental caries in individuals with SCD has been attributed to the presence of enamel hypomineralization. The hypomineralization of the enamel matrix, due to metabolic and hormonal disturbances, would increase the risk of dental caries in these individuals [9]. Other explanations were a lower priority in seeking dental care, especially among individuals with a lower income, and the reluctance of dentists to treat these individuals due of fear of trans- and postoperative complications [10].

The greatest risk of developing caries is also related to frequent hospitalizations due to health complications associated with greater consumption of medication, like antibiotics containing sucrose [11, 12]. On the other hand, children younger than six years old who were taking daily penicillin were reported to have decreased colonization of *Streptococcus mutans* and, therefore, a lower caries experience when compared to healthy individuals who were not taking antibiotics. This difference, however, only existed as long as penicillin was administered [8]. Matos et al. [13] observed a similar caries prevalence and counts of lactobacilli and streptococci in SCD children compared to healthy controls. However, the controls showed a lower salivary buffering capacity. Income significantly influenced the dmft index, when caries prevalence and socioeconomic factors was investigated in 160 SCD patients, aged 3 to 12 years in Recife, Brazil [12].

Regardless of the impact of SCD on dental tissues, the oral health of these individuals is essential to prevent dental infections that could precipitate a vaso-occlusive crisis [14] or act as a bacterial source for development of osteomyelitis of the mandible, which has lost its blood supply [15]. The aim of this study was to investigate the experience of dental caries in a Brazilian pediatric population with SCD, analyzing possible associations with the history and severity of the SCD, socio-economic factors, and their impact on oral health-related quality of life (OHRQoL).

## Methods

The study was conducted in the city of Belo Horizonte, capital of the State of Minas Gerais in southeast Brazil. The study sample was made up of children with sickle cell disease, resident in the metropolitan region of Belo Horizonte, from ages 8 to 14 years old, sampled from the patient registry of the Referral Center for Hematology and Transfusion Services in the State of Minas Gerais (Hemominas-MG). The Human Ethics Committee of Hemominas, and the Dental School of the Federal University of Minas Gerais approved this study under the following protocols 289 and CEP225.951. Written informed consent was obtained from the parents or guardians and the participants of this study.

### Sample characteristics and study design

The investigation was designed as a cross-sectional study. A total of 491 children participated in the study. The participants were divided into two groups: a group with SCD consisting of 106 recruited patients with SCD treated at the Hemominas (56 younger children aged 8–10 years and 50 teens aged 11–14 years), and a comparison group comprising of 385 randomly-selected, healthy children (205 healthy younger children aged 8–10 years and 180 healthy teens aged 11–14 years).

Our sample size was calculated from the expected standard deviations of the quality of life scales to evaluate oral health-related quality of life (OHRQoL) CPQ_8–10_ (10.7) and CPQ_11–14_ (10.1) from a pilot study. The pilot was conducted among a random sample of 34 younger children and 35 teens with SCD in Hemominas-MG. We assumed five points of difference in quality of life scores of SCD patients and a 95 % confidence interval (95% CI). The sample size required was 51 children with SCD and 45 teens with SCD. However 56 younger children with SCD and 50 teens with SCD participated in the study, representing the totality of children with SCD listed at the patient registry in Hemominas, who met the eligibility criteria for the study. We select 180 healthy teens and 205 healthy younger children who were enrolled at the same school and in the same class those SCD children, matched by age and sex. The option to match one case per at least three controls was based on the fact that healthy controls could be less motivated than individuals in a health-care setting [16].

Eligibility criteria for inclusion in the SCD group were as follows: patients aged 8 to 14 years, a diagnosis of SCD HbSS in their medical records, not suffering from a painful crisis at the time of the survey, no medical conditions other than SCD, no emergency dental appointment in the past three months, and did not have an intellectual disability. Eligibility for the healthy control group included: No organic, physiologic, or psychiatric disturbance, no intellectual disability, and no emergency dental appointment in the past three months.

### Training and calibration exercise

The research team was made up of a dentist and four dental researchers. The calibration exercise consisted of two steps. The first step involved a discussion of the criteria established for the diagnosis of each oral health condition through an analysis of 30 photographs. Photographs for dental caries experience included all possible classifications used in this study. A specialist in pediatric dentistry coordinated this step. The next step was performed at Hemominas and consisted of the examination of 10 children aged 10–14 years. The exams were performed on two separate occasions with a one-week interval between sessions. Data analysis involved the calculation of Kappa coefficients (K = 0.89 for interexaminer agreement and K = 0.87 for intraexaminer agreement).

### Data collection

Interviews and dental exams were carried out in private rooms at Hemominas and schools during the day, under natural light.

A qualified dentist performed an intra-oral exam on each patient using a disposable mirror, CPI probes and gauzes. For the examination of the oral condition, we used an exam sheet to determine the DMFT/dmft indexes (separately for permanent teeth and deciduous teeth) and bleeding on probing according to World Health Organization (WHO) criteria [17]. During the clinical examination, the examiner wore personal protective equipment (PPE) that met biosecurity standards.

Data about individual characteristics, socio-economic characteristics and factors related to the disease were collected through direct questioning of the parents or guardians. History of clinical severity was assessed for SCD patients through direct questioning about their frequency of episodes of pain and hospitalization and duration of symptoms, number of associated comorbid conditions, presence of ulcers, and if he (she) is being treated with a blood transfusion. The clinical severity was converted into a score from the sum of the factors related to disease reported by the parents.

A separate researcher (based in a dental research department) read the questions about Oral Health Related Quality of Life (OHRQoL) to respondents, leaving them to mark the answer sheet. For OHRQoL, the Brazilian versions of the Child Perceptions Questionnaires were used for younger children ages 8–10 years (CPQ_8–10_) [18] and for teenagers ages 11–14 years (CPQ_11–14_ short-form version) [19]. CPQ items are distributed into four domains: oral symptoms, functional limitations, emotional well-being and social well-being. A scale was used with the response options of: never = 0; once or twice = 1; sometimes = 2; often = 3 and very often = 4. A high score indicated a more negative impact on OHRQoL. The CPQ_8–10_ is composed of 25 questions and the short-form version of CPQ_11–14_ is composed of 16 questions. The minimum possible score is zero, and the maximum score is 100 for the CPQ_8–10_, and range 0–64 points for the short –form of CPQ_11–14_.

### Statistical analysis

The program Epi info™7 was used for double data entry . Statistical analysis was performed using the Statistical Package for the Social Sciences (SPSS version 20). Descriptive and univariate analyses were performed separately for SCD and healthy children (SCD and controls). The responses to categorical questions by group were compared using either a chi-square test or Fisher`s exact test for contingency tables with small cell counts. The nonparametric Mann Whitney test was used to compare medians of continuous variables between SCD and control groups.

Using the Chi-square test we checked the association between dental caries (No/Yes) and independent variables (individual characteristics, factors related to the disease, socio-economic factors and oral conditions) among SCD patients. In multivariable linear regression analyses of DMFT, we controlled the age and gender at the time of interview.

## Results

The data collection from patients with SCD was performed in Hemominas from January to July of 2012. In order to collect healthy peers, 100 schools were visited from August 2012 to December 2013. All of them were from public schools, and classified as low socioeconomic status (SES).

The response rate was 100 % in both case and comparison groups. In the SCD group there were 106 children: 56 younger children (31 boys and 25 girls) with an average age of 8.9 (SD = 0.87) years and 50 teens (30 boys and 20 girls) with an average age of 12.0 (SD = 1.08) years. There were 385 healthy school children in the comparison group. In this group, there were 205 younger children (112 boys and 93 girls) with an average age of 8.9 (SD = 0.8) years and 180 teens (106 boys and 74 girls) with an average age of 11.9 (SD = 1.0) years (Tables 1 and 2). At least three children who were in the same class as the children with SCD were recruited as controls.

**Table 1:** Descriptive and comparative analysis per individual characteristics, factors related to the disease, socio-economic factors, oral conditions, and negative impact in OHRQoL between younger children (8–10 years) with SCD and control group

	Children SCD N (%)	Children control-group N (%)
*Individual characteristics*		
Gender		
Male	31 (55.3)	112 (54.6)
Female	25 (44.7)	93 (45.4)
Child living with biological parents		
No	21 (41.1)	46 (29.0)
Yes	30 (58.9)	113 (71.0)
*Factors related to the disease*		
Religiosity	6 (10.7)	
No	6 (10.7)	
Sometimes	7 (12.5)	
Frequently	43 (76.8)	
Race		
White	9 (16)	
Black	19 (34)	
Mix	28 (50)	
Age of diagnostic SCD		
<7 months old	52 (95.5)	
7 months - 3 years	3 (5.0)	
*Socio-economic factors*		
Home overcrowding		
<2 people/room	32 (57.0)	101 (49.3)
>2 people/room	24 (43.0)	104 (50.7)
Mother education**		
≤8 years	44 (86.0)	99 (66.5)
>8 years	7 (14.0)	57 (36.5)
Father education**		
≤8 years	35 (83.0)	89 (60.5)
>8 years	7 (17.0)	58 (39.5)
Own house		
No	32 (63.0)	100 (62.9)
Yes	19 (37.0)	59 (37.1)
Own car**		
No	47 (92.0)	117 (73.6)
Yes	4 (8.0)	42 (26.4)
Family income U$/month (mean/SD)	584.4 (245.2)	615.8 (274.7)
*Oral conditions*		
DMFT/dmft (mean/SD)*	1.3 (2.1)	1.8 (2.0)
Decayed (mean/SD)	0.9 (1.8)	1.1 (1.5)
Missing (mean/SD)	0	0.2 (0.6)
Filled (mean/SD)	0.4 (1.2)	0.5 (1.0)
Gingival bleeding (n (%)		
No	47 (84.0)	182 (88.8)
Yes	9 (16.0)	23 (11.2)

*p < .05; **p < .01; Chi-square test, Mann–Whitney test or Fisher's exact test

**Table 2:** Mean and standard deviation (SD) of the CPQ_8–10_ subscales for younger children with SCD and controls (8–10 years)

Domains - CPQ_8–10_	SCD children mean (SD) *N* = 56	Controls children mean (SD) *N* = 205
Oral symptoms	27.68 (17.86)	29.37 (18.82)
Functional limitations	14.11 (15.73)	17.78 (18.96)
Emotional well-being	16.79 (22.55)	19.68 (23.48)
Social well-being	7.23 (8.95)	11.46 (15.56)
Overall CPQ_8–10_	14.51 (12.0)	17.95 (15.0)

In the group of younger children, there was a verified significant difference in the dmft/DMFT, fathers´/mothers´ education, and car ownership among SCD patients and controls (Table 1). The mean overall scores of CPQ_8–10_ in younger children with SCD and their controls were respectively 14.5 (±12) and 17.9 (±15). Table 2 shows the mean of responses to the domains on the CPQ_8–10_. There was no difference in the mean overall score of CPQ _8–10_ among SCD patients and controls (Table 1).

There was no significant difference in mean dmft/DMFT among the teenagers with SCD and their controls (Table 3). Teens with SCD were less likely to be living with both biological parents compared to healthy controls (*p* < .001) and the family income (U$/month) was higher in SCD teens group (*p* < .001). Both maternal and paternal schooling was higher in the SCD teen group (*p* < .001). In the SCD group, only the adolescents had higher prevalence of gingival bleeding than their healthy peers (p < .001) (Table 3). There was no difference in the mean overall score of CPQ _11–14_ among SCD teenagers and healthy peers. The mean scores of the domains of the CPQ _11–14_ are presented in Table 4. All participants completed the CPQ8-10 or CPQ11-14. There were no missing answers of the participants in the CPQ.

**Table 3:** Descriptive and comparative analysis per individual characteristics, factors related to the disease, socio-economic factors, oral conditions, and negative impact in OHRQoL between teens with SCD and control group

	Teens SCD N (%)	Teens control-group N (%)
Individual characteristics		
Gender		
Male	30 (60.0)	106 (58.9)
Female	20 (40.0)	74 (41.1)
Child living with biological parents***		
No	30 (73.0)	56 (38.9)
Yes	11 (27.0)	88 (61.1)
Factors related to the disease		
Religiosity		
No	9 (18.0)	
Sometimes	14 (28.0)	
Frequently	27 (54.0)	
Race		
White	6 (12.0)	
Black	19 (38.0)	
Mix	25 (50.0	
Age of diagnostic SCD		
<7 months old	47 (94.0)	
7 months - 3 years	3 (6.0)	
Socio-economic factors		
Home overcrowding		
<2 people/room	25 (64.0)	90 (62.0)
>2 people/room	14 (36.0)	55 (38.0)
Mother education***		
≤8 years	22 (58.0)	132 (91.7)
>8 years	16 (42.0)	12. (8.3)
Father education***		
≤8 years	14 (61.0)	120 (83.3)
>8 years	9 (39.0)	24 (16.7)
Own house		
No	31 (76.0)	91 (85.9)
Yes	10 (24.0)	53 (14.1)
Own car		
No	40 (98.0)	128 (88.9)
Yes	2 (1.0)	18 (11.1)
Family income U$/month (mean/SD)***	1463 (671)	628 (257)
Oral conditions		
DMFT/dmft (mean/SD)*	1.5 (1.9)	2.1 (2.8)
Decayed (mean/SD)	1.0 (1.4)	1.0 (1.7)
Missing (mean/SD)	0.1 (0.6)	0.2 (0.7)
Filled (mean/SD)	0.4 (0.8)	0.8 (1.7)
Gingival bleeding***		
No	23 (46.0)	152 (84.4)
Yes	27 (54.0)	28 (15.6)

*p < .05; **p < .01; Chi-square test, Mann–Whitney test or Fisher's exact test

**Table 4:** Mean and standard deviation (SD) of the CPQ_11–14_ subscales for SCD teens and controls (11–14 years)

Domains - CPQ_11–14_	SCD teens mean(SD) *N* = 50	Controls teens mean (SD) *N* = 180
Oral symptoms	5.38 (3.40)	4.89 (2.82)
Functional limitations	4.68 (3.62)	3.33 (2.96)
Emotional well-being	3.22 (3.23)	3.71 (3.95)
Social well-being	2.78 (3.49)	2.52 (3.43)
Overall CPQ_11–14_	16.06 (11.65)	14.37 (9.73)

Table 5 shows the results combining the SCD patients and healthy controls. The linear regressions explore the factors related to dmft/DMFT in younger children and teenagers particular to the factor of having SCD. In both younger children and teenagers, diagnosis of SCD was not associated with higher dmft/DMFT indexes. In fact, the only statistically significant determinant of dmft/DMFT in the overall sample was lower family income among the younger children’s group (*p* < .01).

**Table 5:** Beta coefficients of DMFT in children and teens (SCD and control groups) by individual characteristics, factor related to the disease, socio-economic factors, oral conditions

DMFT *(Decayed, Missing and Filled Teeth index)*	Children SCD and Control group		Teens SCD and Control-group	
Predictors	β	SE	β	SE
Age	0.07	0.16	0.20	0.15
Gender boy	0.23	0.27	0.47	0.31
Child living with both biol parents	−0.05	0.34	−0.53	0.36
SCD	0.18	0.34	0.97	0.67
Family income (U$/month)	−0.0011	0.0004**	0.00055	0.00057
Home overcrowding (>2 people/room)	0.14	0.35	0.08	0.32
Mother education	0.14	0.32	−0.41	0.50
Father education	−0.38	0.32	0.38	0.42
Own House	−0.26	0.33	0.18	0.33
Own Car	0.58	0.35	−0.31	0.48
Gingival bleeding	0.50	0.38	0.28	0.37

Children *n* = 261; Teens *n* = 230, β = Beta coeficiente, SE = standard error *p < .05, ^**^ *p < .01*

Table 6 presents the findings restricted to SCD patients. The socioeconomic variables of income and the level of schooling of the father and mother were not associated with dental caries experience among SCD patients. Only the severity of SCD was associated with higher dmft/DMFT (*p* = 0.03).

**Table 6:** Beta coefficients of dmft/DMFT in all patients with SCD by individual characteristics, factors related to the disease, socio-economic factors, and oral conditions (n = 106)

DMFT (Decayed, Missing and Filled Teeth index)	SCD patients	
Predictors	β	SE
Individual characteristics		
Age	0.37	0.20
Gender boy	0.18	0.48
Child living with both biol parents	0.18	0.54
Factor related to the disease		
Diag.age	0.23	1.93
SCD Severity^a^*	0.28	0.12
Socio-economic factors		
Family income (U$/month)	−0.0000590	0.0006535
Home overcrowding (>2 people/room)	0.20	0.54
Mother education	0.16	0.66
Father education	0.82	0.60
Own House	0.67	0.54
Own Car	0.95	0.90
Oral conditions		
Gingival bleeding	0.31	0.61
DAI	0.014	0.027

*n* = 106, SE = standard error *p < .05

^a^SCD Severity: sum of episodes of pain and hospitalization and time occurred (for about a month), number of associated comorbid conditions, presence of ulcers, and if he(she) is being treated with blood transfusion

## Discussion

The present investigation compared the occurrence of dental caries among children and teens with and without SCD. There was a low occurrence of dental caries in children with SCD in comparison to their controls. Among teens, we found no statistical differences when compared to controls in their caries experience, measured by the DMFT index, or the prevalence of untreated caries observed by the decayed component. Our result is similar to that observed in a study of 54 American teenagers with a mean age of 14 years, with sickle cell disease genotypes HbSS and HbSC [6]. The mean DMFT found in the afore mentioned study was 1.94 (2.7) in the SCD group and 2.96 (4.10) in the control group. However, considering the 2 genotypes separately, a significant difference was seen only in teenagers with SCD HbSC (*p* < 0.2). The lack of a significant difference in caries experience between American SCD teenagers HbSS and healthy controls was explained, in part, as a large exposure to fluoride through the drinking water, since children with SCD need to pay attention to hydration when preventing pain crisis [6].

In our results, the minor occurrence of dental caries in the children group was attributed to their receipts of major health care surveillance provided via access to free dental treatment at Hemominas - MG, Brazil. The literature shows a great resilience capacity of children`s families with chronic diseases including sickle cell disease [20, 21]. Mothers of children with SCD often leave their jobs to devote themselves to the child's health needs [20, 21]. Many parents incorporate the role of caregiver or manager for the child´s disease in its own sense of life [20, 21]. In this manner, it is suggested that the SCD children`s health care from their parents had influenced the lower caries experience of their children.

We attribute the lack of significant differences in caries experience between SCD teenagers and the control group to the age of adolescents. The behavior of older children may differ from that of younger children. Teenagers can see the supervision of their parents as a threat to their growing desire of independence, resulting in a resistance to the appropriate behaviors to health [21, 22]. This independence of adolescents with SCD would result in less attention to preventative measures such oral hygiene and a healthy diet, making adolescents with SCD as susceptible to dental caries as their control group.

The experience of dental caries among children with SCD and their peers (without SDC) was similar to the experience observed among 160 children with SCD aged 3 to 12 years in Recife, Brazil [12]. Among children in Recife, there was an average DMFT score of 1.5 [12]. This result is close to what we found. However, comparing our results with data from a national survey carried out in Brazil (dmft = 2.43 and DMFT = 2.07) [23], our results reflected better oral health. Comparing the results of this study with those observed in research conducted with Indian SCD children, there was a higher dental caries experience between Indian SCD children aged 3 to 15 years (Hb SS and Beta thalassemia) in comparison with controls [24]. We believe that one reason for the discrepancy in their findings and our results may be due to differences in the oral health care systems between India and Brazil. In the Brazilian context, oral health care for pediatric SCD patients is provided free of charge by the government. In contrast, In India, the development of appropriate models of health for the diagnosis and follow-up of patients presents a challenge that should not be underestimated [25]. In this case, the characteristics of the Brazilian health system could break the link between socioeconomic conditions and health. Another possible explanation is that our controls were selected from the school friends of SCD patients. There is homophily in social networks [26–28]. This could have resulted in greater similarity of dmft/DMFT scores between patients and their controls (compared to a truly random control population).

Dental caries is a multi-factor disease and controlling factors can be challenging. In our regression analyses, low family income was shown to be a significant associated factor in younger children. Socioeconomic factors have been previously shown to be a robust predictor of caries risk. Lower income may be associated with lower health literacy (concerning oral hygiene practices) as well as lower access to preventive health care information [29, 30]. Our results agree with previous studies showing that income can be considered a co-factor in the relationship between SCD and oral health [10, 12].

Our study revealed a higher frequency of gingival bleeding in adolescents with SCD when compared to the control group. This result differs from that observed in a study of Brazilians between 16 and 68 years of age [10]. According to that study, SCD does not predispose an individual to periodontal disease [10]. The comparison of these studies requires caution since there is no difference in the age of the participants. In the present study, we believe that the association between SCD and bleeding gums can be attributed to the resistance of the appropriate behaviors to health [21], resulting in inadequate oral hygiene, which is a common risk factor for dental caries and periodontal disease.

There was no difference in OHRQoL among participants with SCD and controls. A similar result was found in SCD teenagers and controls in Ohio (USA) [6]. As described by these authors, the lack of association may be explained by the patient selection bias. The present study only included participants who were not suffering from a painful crisis at the time of the survey, the medical conditions other than SCD, and in emergency dental appointments in the past three months. These facts may have led to the evaluation of only patients in good health, masking the OHRQoL during the acute pain that is a characteristic of SCD.

We would have expected parental education to be a strong predictor of caries. Less educated parents have less health literacy. However, considering the schooling level of the fathers and mothers of all the children in our study, we observed that 76 % of parents had completed up to eight years of study. It is a very homogeneous sample for the low level of education. This could be a reason for not having found a relationship between the prevalence of caries in children and the level of parental education, either in univariate or multivariate analysis. This result agrees with the study of 160 Brazilian children with SCD aged 3–12 years in Recife, Brazil [12]. There was no significant relationship between parental education level and dmft/DMFT index, or any of the variables examined: decayed, missing and filled teeth [12]. Over half of the parents (56.3 %) had not completed their elementary education, 18.1 % had completed elementary school, and 18.1 % had completed high school [12].

In the present study, the disease's clinical severity was associated with dental caries in children suffering from SCD. Disease's clinical severity is associated with vaso-occlusion within the vasculature of the pulpal tissue that may account for the pain experienced by SCD sufferers, and that the same infarction process that afflicts other organs in the body may also affect the dental tissue [31]. Other studies examined histological changes in the dental tissue with regard to SCD and disease's clinical severity. Sickle-shaped cells were observed in the vessels of the pulp of teeth from patients 24 to 96 h after an acute sickle cell crisis attack [32]. The authors concluded that significant pathological changes were observed in the dentin and pulp and suggested that they were most likely due to stasis of the sickle cells in the capillaries, which created a hypoxic situation, causing tissue infarction. The authors stated that the changes observed were not pathognomonic, but that findings suggest that the sickle cell disease's clinical severity has a higher susceptibility to poor oral health [32, 33]. The knowledge of the association between dental caries and the severity of SCD allows dentists to gain a greater understanding of the problem and the role of pediatric dentists in the overall health of children.

The main limitation of this study was that we examined a specific population from one city in southern Brazil. Accordingly, our findings should be interpreted in the light of external generalization. Prospective studies which have larger and more diverse samples involving hospitalized patients, should complement our data and would provide a better understanding of the relationship between the severity of SCD and oral health. Dental radiographs were not obtained; thus, carious lesions that can only be detected radiographically were missed. Moreover consumption of sugar was not assessed. In terms of the strengths of our study, we conducted oral examinations on every subject, and components of the caries burden index (dmft/DMFT) were separated, making it possible to differentiate active disease from treated disease. We included a matched control group consisting of healthy peers. Lastly, the high degree of reproducibility of the diagnoses, as measured by the kappa statistics, contributed to the internal validity of the study.

## Conclusion

In conclusion, children with SCD had less experience of dental caries than controls (without SCD). The occurrence of dental caries among teenagers with SCD was similar to their controls. The results reinforce the importance of care related to oral health among children and emphasize the need to implement appropriate preventive measures for these patients taking into account factors that were associated with caries. These measures, in addition to preventing the occurrence of decay, are fundamental to avoid complications in the general health of children with SCD. Therefore, dentists have an important role in preventing health complications in patients with SCD and providing a better quality of life for them.

## Abbreviations

SCD: Sickle Cell Disease
WHO: World Health Organization
PPE: Personal Protective Equipment
OHRQoL: Oral Health-related Quality of Life
CPQ: Child Perceptions Questionnaire
SES: Socioeconomic Status
QoL: Quality of Life
HbSS: Genotype Hemoglobin SS (Homozigoze of hemolobin S)
HbSC: Genotype Hemoglobin SC
